# Tobacco use among urban slum dwellers attending a cancer screening clinic in the National Capital Region of India: a cross-sectional study

**DOI:** 10.3332/ecancer.2021.1230

**Published:** 2021-05-11

**Authors:** Suzanne T Nethan, Dhirendra N. Sinha, Ashwini Kedar, Vipin Kumar, Shashi Sharma, Roopa Hariprasad, Ravi Mehrotra

**Affiliations:** 1 School of Preventive Oncology, A/27, Anandpuri, West Boring Canal Road, Patna 800001, India; 2 National Viral Hepatitis Control Program, Ministry of Health & Family Welfare, Nirman Bhawan, Near Udyog Bhawan Metro Station, Maulana Azad Rd, Delhi 110011, India; 3 Nippon Data System Ltd., B-14, Sector 8, Noida 201301, India; 4 Senior Biostatistician, C58/25, B902, Jagdamba Apartments, Sector 62, Noida 201307, India; 5 Division of Clinical Oncology, Indian Council of Medical Research-National Institute of Cancer Prevention & Research (ICMR-NICPR), I-7 Sector 39, Noida 201301, India; 6 Chief Executive Officer, ICMR – India Cancer Research Consortium, IRCS Building, Red Cross Road, Delhi 110003, India

**Keywords:** urban slums, Noida, India, tobacco, oral cancer

## Abstract

**Background:**

Tobacco consumption in India varies based on the place of residence (urban/rural). Minimal, exclusive information exists regarding the same for ‘urban slum’ dwellers. The current study determines the tobacco use pattern among such individuals in Noida, Uttar Pradesh (India).

**Methods:**

A cross-sectional study was conducted among the urban slum residents visiting the institutional clinic between December 2016 and June 2019. Apart from tobacco history, routine recording of the basic demographic details and oral visual examination was carried out for the participants. For categorical data, the percentage of different parameters was calculated and for quantitative data, descriptive statistics were calculated. Chi-square or Fisher’s exact tests were employed to determine the association between the two categorical variables. To find the strength of association between tobacco use and the socio-demographic factors, univariate and multivariable binary logistic regression was used.

**Results:**

Among 2,043 urban slum respondents (602 male, 1441 female), 15.0% (n = 308) currently consumed tobacco. The majority were smokeless tobacco (SLT) users (among both males and females). Among males, khaini (42.1%) and gutkha (32.5%) and among females gul (36.1%) were the most widely used tobacco products.

**Conclusion:**

The majority of the Noida urban slum population attending the screening clinic consumed SLT. Gender variation in the tobacco form and product-specific consumption patterns indicates that the undertaking of urban slums-specific surveys is essential. Tobacco control programmes must incorporate appropriate strategies addressing such subgroups of tobacco users.

## Background

Tobacco, in any form, is the primary aetiological factor for the development of oral cancer and yet the most preventable cause of death [1]. Tobacco use is also a non-communicable disease risk factor that is responsible for 70% of mortality in India [2]. A myriad variety of both smoked and smokeless tobacco (SLT) products are available in the Indian market [3]. Bidi is the most commonly smoked form (8% adult users), while khaini is the most common smokeless form of tobacco (11% adult users) consumed in India [4]. By 2025, 27.2% of the total cancer burden is predicted to comprise tobacco-related cancers [5]. India contributes to one-third of the global oral cancer burden [6].

Rapid and large-scale mobilisation of rural residents to urban areas for employment, in many low and middle-income countries, has resulted in the urbanisation of poverty. Such a phenomenon and deprivation of available services often leads to residential crowding, and social fragmentation and exclusion, referred to as a ‘slum/urban slum’ [7]. Urban slum dwellers have poor health indicators either due to the lack of access to affordable healthcare or due to prevailing poor sanitary conditions and lack of health education. Due to the various socio-economic issues, they are also more prone to developing addictions, such as tobacco use and other substance abuse. The 2011 census reported of 377 million residents in the urban slums of India which is predicted to rise to 535 million by 2026 [8, 9]. The estimated number of individuals living in the urban slums of Noida (Uttar Pradesh, UP) is 49,407 [10].

The previously held national surveys, such as the Global Adult Tobacco Surveys (GATS) [4] and the National Family Health Surveys (NFHS) [11], have discussed the tobacco consumption patterns among both rural and urban inhabitants of the Indian states and union territories. However, the current information regarding tobacco consumption patterns of urban slum dwellers (including those in UP, where this study was carried out) [12–23], and if they are any different or similar to consumption patterns among the other two locational groups (urban and rural) of tobacco users, is inadequate. Even the aforementioned national-level surveys have either not reported this information separately (in GATS the information is provided for urban residents in toto, not exclusively for the urban slum dwellers) [4] or only in one round (NFHS 3, 2005-06) [24].

However, there is a paucity of studies in the literature about the prevalence, pattern of tobacco use and the factors determining tobacco use among urban slum dwellers. Hence, the present study was conducted to determine the pattern of tobacco consumption among residents of urban slums in Noida (UP) attending our cancer screening clinic.

## Methods

### Study design

This study used the cross-sectional study design.

### Setting

The study was conducted at the cancer screening clinic at our organisation in Noida, UP (India). At the clinic, regular screening of people is conducted for hypertension (HT), diabetes mellitus (DM), oral cancer and oral potentially malignant disorders (through oral visual examination), breast cancer (through clinical breast examination) and cervical cancer and pre-cancer (through Papanicolaou and visual inspection with acetic acid tests). Systematic population-based screening and opportunistic screening are conducted at the facility. People are referred from nearby facilities for screening of either breast, oral, or cervical cancer. These people were made aware of these three common cancers and were then motivated to get themselves screened.

Systematic population-based screening is carried out with the help of primary healthcare workers—accredited social health activists who motivate the eligible population in the community for screening.

### Selection of participants

The study participants were selected using a convenient sampling method. People were made aware of the importance of screening and about the diseases for which they are being screened. People who agreed to screening were included in the study.

All people in the age group 30–65 years and those having a history of tobacco use in other age groups (as per the operational guidelines) [25] and residing in urban slums were included in the study.

A total of 2043 urban slum dwellers were included in the study. The study was conducted from December 2016 to June 2019.

### Operational definitions

Slum dweller—a person living in an urban slum for 6 months or more [26].

Smoker—a consumer of cigarettes, bidis, hookah, hand-rolled cigarettes, pipes, cigars/cheroots/cigarillos and any other reported smoked tobacco products [4].

SLT user—consumption included khaini, gutkha, betel quid (with tobacco) and any other reported SLT products which are edible, applied orally or inhaled; non-tobacco areca nut-based products were also included under this category [4].

Current tobacco user—any person giving a history of tobacco use on the day of the interview was termed as a current tobacco user [4].

Former/past tobacco user—any person who stated to have quit tobacco use on the day of the interview was termed as former/past tobacco use [4].

### Data collection and processing

Following written informed consent, a detailed history of tobacco use was elicited from all the study participants.

The participants were interviewed using the pre-designed online clinical patient record form wherein their demographic details and tobacco use history, if present (current status, type of tobacco product used, amount, duration and frequency of tobacco use), were recorded. To ensure accurate data entry and to avoid missing data, regular monitoring of data was done utilising inbuilt data checks in the online software.

A number of potential sources of bias were noted and appropriate measures were undertaken to address these. Tobacco consumption is still quite a taboo among women who are thus not always comfortable revealing such information. Owing to a majority of females, and thus the impending information bias, a conducive, comfortable environment was developed for the participant by first building a rapport, followed by gradual probing regarding their tobacco history. Two different investigators elicited information by framing questions in slightly different ways to overcome recall bias.

### Statistical methods used

The collected data were entered into an excel file, and analysed using the Statistical Package for the Social Sciences version 21, and OpenEpi open-source software. For categorical data, the percentage of different parameters was calculated and for quantitative data, descriptive statistics were calculated (mean, standard deviation and range). Chi-square or Fisher’s exact test was used to find an association between two categorical variables. To find the strength of association between tobacco use and the socio-demographic factors, univariate and multivariable binary logistic regression was used. Socio-demographic variables which were considered for analysis were dichotomised. For example, the education variable was split into two: literate (those with some formal education including those who could only read and write) and illiterate (those who had not received any formal education and could not read and write). For income stratification, the BG Prasad classification [27] was used. The higher side income of the middle class was taken as the cut-off for dichotomisation of the variable. A statistical significance was established at a *p*-value < 0.05.

### Ethical guidelines followed by the investigators

The study was approved by the institutional ethical committee (approval number: ICPO/IEC/2014). Written informed consent was taken from all participants who were involved in the study.

## Results

The data obtained from 2043 participants were analysed.

### Socio-demographic details

All the socio-demographic details of the study participants are given in Table 1.

The participants were aged between 16 and 85 years [average age = 36.3 (± 10.9) years]. The majority were married (92.5%), homemakers (majority female population)/students (64.6%) and illiterate (36.2%), with a monthly per capita income of ≤ 3,500 of Indian rupees among the majority of the study participants (Table 1).

### Prevalence and pattern of tobacco use

**Figure 1.**
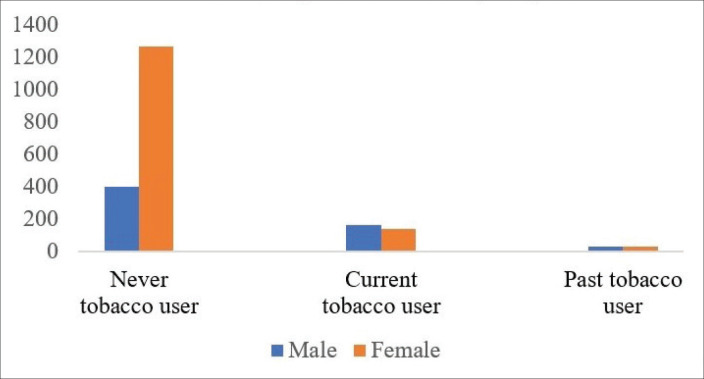
Tobacco use among male and female participants (n = 2043).

Among the 2,043 urban slum dwellers, 308 (15.06%) individuals were current tobacco users (166 males and 142 females) and 64 (3.1%) individuals were former tobacco users, while 1,671 (81.8%) individuals never consumed tobacco (Figure 1). Majority of the current tobacco users consumed SLT (68.8%), followed by tobacco smoking (16.9%), while 14.3% of individuals consumed both (dual users). Among both genders, females showed a similar pattern of consumption; however, among males, SLT use was predominant, followed by dual tobacco use and smoked tobacco use.

The pattern of current tobacco use varied with regard to different socio-demographic factors; a significant association was found between the type of tobacco use (smoked, smokeless or both) with age, gender and education (Table 2).

### Types of tobacco products consumption—current tobacco users

As summarised in Table 3, the most common type of tobacco products used among the current tobacco users were khaini (28.5%), gutkha (23.0%), bidi (22.8%) and gulmanjan/gul (21.1%). Bidi was the preferred form of smoked tobacco among both genders. However, a gender variation was noted in the consumption of SLT products, with khaini (42.1%) and gutkha (32.5%) use being most common among males, while gulmanjan/gul (36.1%) was the most widely consumed product by females, followed by non-tobacco, areca nut-based products (betel quid pan masala and supari) (19.1%). The type of tobacco products used was strongly associated with gender (Table 3).

### Determinants of tobacco use for current and past tobacco users

Table 4 shows age greater than 45 years, being male, having education till middle school or below (includes illiterates) and being single/widowed/separated/divorced as significant determinants for current tobacco use. The odds of current tobacco use were 3.85 among males as compared to females, 1.49 among individuals aged 45 years and above as compared to those younger than 45 years, 1.58 among those who were illiterate or had education till middle school and 2.11 among those who were single/separated/divorced/widowed as compared to married individuals. The main determinant for former tobacco users was being male [odds ratio (OR) = 3.45, *p* = 0.00], as the other socio-demographic factors were not found to affect former tobacco use.

## Discussion

### Socio-demographic profile

Tobacco use was more prevalent among males than females, as seen in the current study and also reported by the GATS-2 (UP) [28] and few other studies conducted in the Indian urban slums [12, 15, 19, 21, 23]. This stark gender variation may be attributed to tobacco consumption being a societal taboo among females in India.

### Prevalence and pattern of tobacco use

The prevalence of current tobacco use among the study participants belonging to the urban slums of Noida (UP), India, was 15%. The same in GATS-2 for India [4] was 28.6% and 35.5% for UP [28]. The prevalence for current tobacco use was lower in the current study which may be due to a skewed sample with a greater number of females. The current tobacco use among males was 27.5% and among female was 9.8%, which was found to be lower when compared to the same in the GATS-2 report [28].

In the current study, the majority consumed SLT (68.8%), followed by smoked tobacco (16.9%) and both (14.3%). This is in line with the GATS-2 survey of UP [28]. Similar findings were noted in a study carried out among the urban slum dwellers of Bengaluru (Karnataka) [18]. However, in studies carried out in the urban slums of Shillong (Meghalaya) [23] and North 24 Parganas (West Bengal) [15], a high percentage of SLT users was followed by an almost similar percentage of smokers and dual users. On the contrary, a majority of dual tobacco users was followed by SLT users, and smokers in the urban slums of Bhopal (Madhya Pradesh) [19], whereas equal percentages of dual tobacco and SLT users, with a relatively lower percentage of smokers, were seen in the Shillong urban slums [22]. Overall, SLT use was more prevalent than smoked tobacco; hence, appropriate policy measures towards its control are essential.

### Types of tobacco products consumption: current tobacco users

In our study, khaini (28.5%) was the most common tobacco product consumed. This was followed by gutkha (23%) consumption. The type of tobacco products consumed overall was similar to that consumed by rural residents of UP as per the GATS-2 for UP [28]. However, the choice of SLT product among the urban residents of UP was different, with gutkha (10.2%) being the product of choice for consumption, followed by khaini (9.1%) [28]. This is suggestive of the retention of the original tobacco consumption practice by the rural natives in spite of migrating to urban areas. Most males consumed khaini (42.1%) and gutkha (32.5%), while gul (36.1%) use was most common among females in the present study.

As mentioned above, certain tobacco products have shown a much higher prevalence of consumption by females, such as gul, including in the current study (most commonly used tobacco product by them). Even the GATS-2 for UP [28] reported gul as the second most widely used tobacco product among women (rural and urban). Multiple studies have reported high consumption of gul among Indian females, such as those conducted in rural Patna [33], at a tertiary care centre in Delhi [34] and in an urban slum of Mumbai [17]. In a study conducted in Bangladesh, another country with indigenous gul use, almost twice the prevalence was reported among females (10%) than males (5.3%) [35]. Orodental pain secondary to dental caries, endodontic lesions or periodontal conditions, such as periodontitis or gingivitis, has been reported as the main reason for the initiation of gul, due to the acute analgesic effect of tobacco [36, 37]. Gul has also been shown to be highly addictive in nature [38], with a reported average frequency of use from 2 up to 50 times per day [17, 39, 40]. Hence, gender-targeted tobacco control advocacy, exclusively for female tobacco users, is the need of the hour.

### Determinants of tobacco use for current and past tobacco users

Age, gender and education showed a significant association with the tobacco form used (smoked, SLT or both), in both the current study and that in the urban slum of Shillong [23]. GATS-2, India [4], also reported an increase in the overall tobacco prevalence with increasing age, low education levels and in males.

Older age and males were significant determinants for tobacco use in our study as well as in the study conducted in the urban slums of Chennai [14], while illiteracy as a significant determinant of tobacco use was reported in the study conducted in Faridabad [13], in addition to ours.

A systematic review showed that SLT use was more common among the lower socio-economic group, less educated and older population [41].

Apart from tobacco-containing areca nut products, plain areca nut-based products (i.e. without tobacco), such as betel quid (without tobacco), pan masala, and supari, were also widely consumed (11.7%) by the participants, especially females (second most widely consumed tobacco product type). The GATS-2 UP [28] also reported a high overall (rural and urban) prevalence of non-tobacco products (pan masala, 7%; betel quid, 12.8%; and areca nut 7.6%). Significant consumption of pan masala was also observed in the urban slums of Shillong [23] and Bengaluru [18]. The chewing of areca nut is an ancient practice ingrained in the Indian culture [29]. Betel leaf and areca nut are an integral part of Hindu ceremonies and a source of livelihood for the many Indian states [30]. Its consumption is socially acceptable, including among both women and children [31]. However, areca nut has been classified as a group 1 carcinogen by the International Agency for Research on Cancer [32], thereby deeming the control and monitoring of its consumption as essential as tobacco.

The Government of India has actively proposed various tobacco control policies; however, their effective, complete implementation is the need of the hour. The patterns of tobacco consumption, across the various strata of the society, i.e. urban, rural and slums, should be continuously assessed, which will in turn help in developing effective, appropriate interventions for the same [42]. Sensitisation and encouraging healthcare providers regarding tobacco cessation assistance are needed [43]. The tobacco cessation programme may be collated with other existing healthcare programmes [42]. Also, education and awareness regarding the adverse health effects due to tobacco consumption need to be spread [43].

### Limitations of this study

The study population may not be a true representation of the community due to the institutional clinic setting and convenience sampling.

However, the study provides important insights into the tobacco use habits of the urban slum dwellers who are usually migrants and are slowly growing in numbers due to the increase in urbanisation. A predominance of females in the study population not only provides essential information regarding their tobacco use patterns but also important cues about their health-seeking behaviour, by virtue of them attending our clinic for accessing breast and cervical cancer screening services.

## Conclusion

The present study is the first to report the prevalence of tobacco use among the urban slum population of Noida (UP), India. SLT is the most widely consumed tobacco form, even in this population attending the clinic, thus reaffirming the importance of SLT control, compared to that of smoked tobacco, in our country. The study brings up the fact that the lower socio-economic group, less educated, males and the older population are more likely to consume tobacco in an urban slum. Although tobacco use is less prevalent among women, they do consume areca nut products and other forms of tobacco, such as ‘gul’, which they consider safe to use. Hence, the National Tobacco Control Programme may focus on these population groups to control tobacco use.

## Conflicts of interest

The authors declare that they have no conflict of interests.

## Funding

This work was supported by the Indian Council of Medical Research [5/13/49/2014/NCD-III].

## Authors’ contributions

Conception or design of the work, acquisition, analysis and interpretation of data: STN, DNS, VK, AK and SS. Drafting the work or revising it critically for important intellectual content: STN, DNS, AK, RH and RM. Final approval of the version published: DNS and RM. Agreement to be accountable for all aspects of the work in ensuring that questions related to the accuracy or integrity of any part of the work are appropriately investigated and resolved: STN, DNS, VK, AK, SS, RH and RM.

## Figures and Tables

**Table 1.** Socio-demographic profile of the urban slum dwellers.

Demographic characteristic		Males (*n* = 602)		Females (*n* = 1,441)		Total (*n* = 2,043)	
		*n*	%	*n*	%	*N*	%
Age (in years)	15–24	60	2.9	149	7.3	209	10.2
	25–34	191	9.3	543	26.6	734	35.9
	35–44	200	9.8	461	22.6	661	32.3
	45–54	98	4.8	207	10.1	305	14.9
	55–64	44	2.1	66	3.2	110	5.3
	65–74	7	0.3	12	0.6	19	0.9
	≥75	2	0.1	3	0.1	5	0.2
Marital status	Married	541	26.5	1350	66.1	1891	92.5
	Divorced	1	0.0	5	0.2	6	0.3
	Single	42	2.1	19	0.9	61	3.0
	Widowed	18	0.9	67	3.3	85	4.2
Occupation	Clerical and support work, services and sales work	17	2.8	6	0.4	23	1.1
	Skilled workers and shop and market sales workers, skilled agricultural and fishery workers, business including retailers	50	8.2	60	4.1	110	5.3
	Elementary occupation	239	39.6	236	16.3	475	23.2
	Unemployed, retired, widowed, chronically ill, permanently disabled	104	17.2	10	0.6	114	5.5
	Student, homemaker	192	31.8	1129	78.2	1321	64.6
Education	College and above	47	2.3	74	3.6	121	5.9
	Intermediate	83	4.1	142	6.9	225	11
	High School	120	5.9	172	8.4	292	14.3
	Middle	85	4.2	241	11.8	326	15.9
	Primary	76	3.7	182	8.9	258	12.6
	Others/read and write	32	1.6	50	2.5	82	4
	Illiterate	159	7.8	580	28.4	739	36.2
Religion	Christian	1	0.01	3	0.1	4	0.2
	Hindu	547	26.8	1305	63.9	1854	90.7
	Muslim	54	2.6	133	6.5	187	9.1
Monthly per capita income (in Indian rupees)^a^	<1,051	40	2.0	67	3.3	107	5.2
	1,051–2,101	244	11.9	502	24.5	746	36.5
	2,102–3,503	184	9	530	26	715	35
	3504–7007	108	5.3	287	14	396	19.4
	>7,008	26	1.3	55	2.7	81	4

a BG Prasad classification 2019 [27]

**Table 2.** Pattern of current tobacco use and its association with socio-demographic factors.

Demographic characteristic	Smoking (*n* = 52, 16.9%)	SLT (*n* = 212, 68.8%)	Dual tobacco use (*n* = 44, 14.3%)	Total (*n* = 308, 15.0%)	Chi-square test (*p* value)
Age (in years)					
Less than 45	29 (9.4)	157 (51)	27 (8.8)	213 (69.2)	8.01 (*p* = 0.02)
Greater than or equal to 45	23 (7.5)	55 (17.9)	17 (5.5)	95 (30.8)	
Gender					
Female	22 (7.1)	110 (35.7)	10 (3.2)	142 (46.1)	12.83 (*p* = 0.00)^a^
Male	30 (9.7)	102 (33.1)	34 (11)	166 (53.9)	
Per capita monthly income (in Indian Rupees)					
>3,501	15 (4.9)	40 (13)	11 (3.6)	66 (21.4)	2.86 (*p* = 0.24)
Less than or equal to 3,500	37 (12)	172 (55.8)	33 (10.7)	242 (78.6)	
Education					
Illiterate	34 (11)	82 (26.6)	13 (14.2)	129 (41.9)	15.45 (*p* = 0.00)^a^
Literate	18 (5.8)	130 (42.2)	31 (10.1)	179 (58.1)	
Marital status					
Married	41 (13.3)	188 (61)	36 (11.7)	265 (86)	4.12 (*p* = 0.13)
Others (single/divorced/separated/widowed)	11 (3.6)	24 (7.8)	8 (2.6)	43 (14)	

a Significant at *p* < 0.05.

**Table 3.** Prevalence of the various tobacco products used among current tobacco users.

Tobacco product	Males (*n* = 166)		Females (*n* = 142)		Total (*n* = 308)		*p*-value
	*N*	%	*n*	%	*N*	%	
**Smoked tobacc**o							
Bidi	44	26.5	26	18.44	70	22.80	*p* = 0.28
Cigarette	18	10.8	5	3.55	23	7.49	
Others	3	1.8	3	1.42	6	1.63	
SLT							
Khaini	70	42.1	18	12.7	88	28.5	
Gutkha	54	32.5	17	12.0	71	23.0	*ϰ*^2^ = 76.94
Betel quid (with tobacco) and other regional/local tobacco preparations (gudhaku)	28	16.8	25	17.7	53	17.2	*p* = <0.00001^a^
Non-tobacco areca nut-based products (betel quid without tobacco, pan masala, supari)	9	5.5	27	19.1	36	11.7	
Gulmanjan/Gul	14	8.4	51	36.1	65	21.1	

a Significant at *p* < 0.05

**Table 4.** Socio-demographic determinants of current and past tobacco use among urban slum participants^a^.

Demographic characteristic	Current tobacco use^b^ crude OR (*p* value)	Current tobacco use^b^ adjusted OR (*p* value)	Past tobacco use^b^ crude OR (*p* value)	Past tobacco use^b^ adjusted OR (*p* value)
Age				
Less than 45	1	1	1	1
Greater than or equal to 45	1.83 (1.40–2.40) *p* = 0.00^c^	1.49 (1.12–1.99) *p* = 0.01^c^	1.49 (0.84–2.62) *p* = 0.17	1.24 (0.69–2.23) *p* = 0.48
Gender				
Female	1	1	1	1
Male	3.68 (2.86–4.73) *p* = 0.00^c^	3.85 (2.97–4.99) *p* = 0.00^c^	3.35 (2.03–5.54) *p* = 0.00^c^	3.45 (2.06–5.76) *p* = 0.00^c^
Per capita monthly income (in Indian Rupees)				
>3,001	1	1	1	1
Less than or equal to 3,000	1.31 (0.99–1.73) *p* = 0.06^c^	1.02 (0.75–1.39) *p* = 0.91	1.40 (0.78–2.53) *p* = 0.26	1.24 (0.65–2.38) *p* = 0.52
Education				
High school and above	1		1	1
Middle school and below (includes illiterates)	1.34 (1.02–1.76) *p* = 0.04^c^	1.58 (1.17–2.13) *p* = 0.003^c^	1.22 (0.70–2.13) *p* = 0.48	1.41 (0.78–2.54) *p* = 0.26
Marital status				
Married	1	1	1	1
Others	2.52 (1.73–3.69) *p* = 0.00^c^	2.11 (1.42–3.14) *p* =0.00^c^	2.22 (1.03–4.79) *p* = 0.04^c^	1.92 (0.88–4.20) *p* = 0.10

OR = odds ratio

a Binary logistic regression analysis

b Reference group is never tobacco user

c Significant at *p* < 0.05
